# TNF inhibits catecholamine production from induced sympathetic neuron-like cells in rheumatoid arthritis and osteoarthritis *in vitro*

**DOI:** 10.1038/s41598-018-27927-8

**Published:** 2018-06-25

**Authors:** Markus Herrmann, Sven Anders, Rainer H. Straub, Zsuzsa Jenei-Lanzl

**Affiliations:** 1 Laboratory of Experimental Rheumatology and Neuroendocrine Immunology, Department of Internal Medicine, University Hospital Regensburg, Regensburg, Germany; 2 Department of Orthopedic Surgery, University Hospital Regensburg, Asklepios Clinic Bad Abbach, Kaiser Karl V Allee 3, 93077 Bad Abbach, Germany; 3 Dr. Rolf M. Schwiete Research Unit for Osteoarthritis, Orthopedic University Hospital Friedrichsheim gGmbH, Frankfurt/Main, Germany

## Abstract

Synovial adipose stem cells (sASC) can be differentiated into catecholamine-expressing sympathetic neuron-like cells to treat experimental arthritis. However, the pro-inflammatory tumor necrosis factor (TNF) is known to be toxic to catecholaminergic cells (see Parkinson disease), and this may prevent anti-inflammatory effects in inflamed tissue. We hypothesized that TNF exhibits inhibitory effects on human differentiated sympathetic tyrosine hydroxylase-positive (TH+) neuron-like cells. For the first time, iTH+ neuron-like sympathetic cells were generated from sACSs of rheumatoid arthritis (RA) and osteoarthritis (OA) synovial tissue. Compared to untreated controls in both OA and RA, TNF-treated iTH+ cells demonstrated a weaker staining of catecholaminergic markers in cell cultures of RA/OA patients, and the amount of produced noradrenaline was markedly lower. These effects were reversed by etanercept. Exposure of iTH+ cells to synovial fluid of RA patients showed similar inhibitory effects. In mixed synovial cells, significant effects of TNF on catecholamine release were observed only in OA. This study shows that TNF inhibits iTH+ synovial cells leading to the decrease of secreted noradrenaline. This might be a reason why discovered newly appearing TH+ cells in the synovium are not able to develop their possible full anti-inflammatory role in arthritis.

## Introduction

In previous studies, newly appearing tyrosine hydroxylase-positive (TH+) catecholamine-producing cells have been detected in synovial tissue of rheumatoid arthritis (RA) and osteoarthritis (OA) patients^1,2^. Catecholamines, such as noradrenaline (NA) are able to mediate anti-inflammatory effects in RA depending on concentration and targeted receptor subtype (it is β2-adrenergic)^3,4^. The anti- inflammatory character of these TH+ cells has been demonstrated *in vivo* by adoptive transfer of generated TH+ cells in the collagen type II-induced arthritis model in mice^5^.

Generation of these TH+ cells might be possible using synovial adipose stem cells (sASC), because mesenchymal stem cells can differentiate into sympathetic neuron-like cells^5–8^. Different cell types, such as fibroblasts, macrophages or B cells have been identified among these spontaneously appearing TH+ cells in RA and OA synovial tissue^1^. Moreover, resident synovial stem cells^9,10^ might differentiate to a catecholaminergic phenotype, given the fact that brain-derived neurotrophic factor, well known to drive differentiation of new catecholaminergic neurons, is present in inflamed joints^11,12^. The question appears whether, or not, the inflammatory environment interferes with TH+ cells.

The cytokine TNF is a major mediator of inflammation, it often starts the chronological cascade of inflammation, and it has also been shown to play a role in neuroinflammatory brain disorders^13–15^. An early study in Parkinson disease showed a reduced TH expression in the CNS of TNF-overexpressing mice^16^. In a rat Parkinson disease model, TNF was toxic to catecholaminergic neurons^17^, which was also confirmed *in vitro*^18–21^. Thus, inhibiting TNF might also influence catecholamine production of these cells.

Neutralizing TNF using anti-TNF strategies is an established therapy option in inflammatory diseases^22,23^. During the last decade, numerous TNF-inhibitors were developed, e.g. etanercept (soluble TNF receptor 2), which has been shown to be neuroprotective in a mouse model of Parkinson disease^24^. TNF inhibitors down-regulate a range of pro-inflammatory cytokines in synoviocytes of RA patients^25,26]^. They ameliorate joint disease in a murine collagen type II-induced arthritis model^27–29^. However, the effect of TNF or/and anti-TNF on anti-inflammatory TH+ catecholamine-producing cells in chronic arthritis is not yet known. If TNF interferes with catecholamine production by inhibiting TH in synovial cells, this would be a proinflammatory signal due to the anti-inflammatory role of catecholamines at high concentrations through β2-adrenergic receptors^3,4^.

Since TH can be upregulated under hypoxic conditions in induced TH+ cells^5^, the phenomenon might be even more evident at low oxygen tension. Most of the studies on TNF inhibitors were limited only to normoxic conditions. Only one study analysing TNF effects on catecholaminergic PC12 cells was performed under hypoxia confirming an influence on TH expression^30^. This is a very important point because inflammation is accompanied by hypoxia^30^, and the milieu in the joint tissue is hypoxic^31^.

Taken all background information together, we hypothesized that we can differentiate sASC into iTH+ catecholamine-producing cells. We also hypothesized that TNF inhibits characteristic features of iTH+ catecholamine-producing cells, whether differentiated from sASC or derived from chronically inflamed synovium. It was further hypothesized that neutralization of TNF results in an enhanced TH expression and catecholamine secretion. Material of patients with RA and OA was in the focus.

## Material and Methods

### Patients

Synovial tissue and synovial fluid was obtained from patients with OA and RA during knee joint replacement surgery (characteristics of patients in Table 1). Diagnosis of RA was based on the established criteria according to the American College of Rheumatology (formerly, the American Rheumatism Association)^32^. Patients were informed about the purpose of the study and gave written consent. The project was approved by the Ethics Committee of the University of Regensburg (Number 13-101-0135). All experiments were performed in accordance with relevant guidelines and regulations.

**Table 1:** Characteristics of patients under study.

	Osteoarthritis	Rheumatoid Arthritis
number	24	16
age, yr	70.5 ± 9.1 [44–81]	65.1 ± 8.8 [51–82]
women/men, n (%)	15/9 (37.5/62.5)	10/6 (62.5/37.5)
C-reactive protein, mg/l	1.8 ± 1.8	5.9 ± 6.1
Medication		
daily prednisolone, mg	0 (0)	6.05 ± 4.8
prednisolone, n (%)	n.a.	11 (68.8)
methotrexate, n (%)	n.a.	6 (37.5)
leflunomide, n (%)	n.a.	2 (12.5)
sulfasalazine, n (%)	n.a.	0 (0)
hydroxychloroquine, n (%)	n.a.	0 (0)
non-steroidal antiinflammatory drugs, n (%)	24 (100)	16 (100)
opioid analgesics, n (%)	2 (8.3)	4 (25)
biologicals, n (%)	n.a.	1 (6.1)

Data are given as means ± SEM, percentages in parentheses, and ranges in brackets.

Abbreviation: n.a., not applicable.

### Human synovial adipose tissue-derived stem cells (sASC)

**Figure 1:**
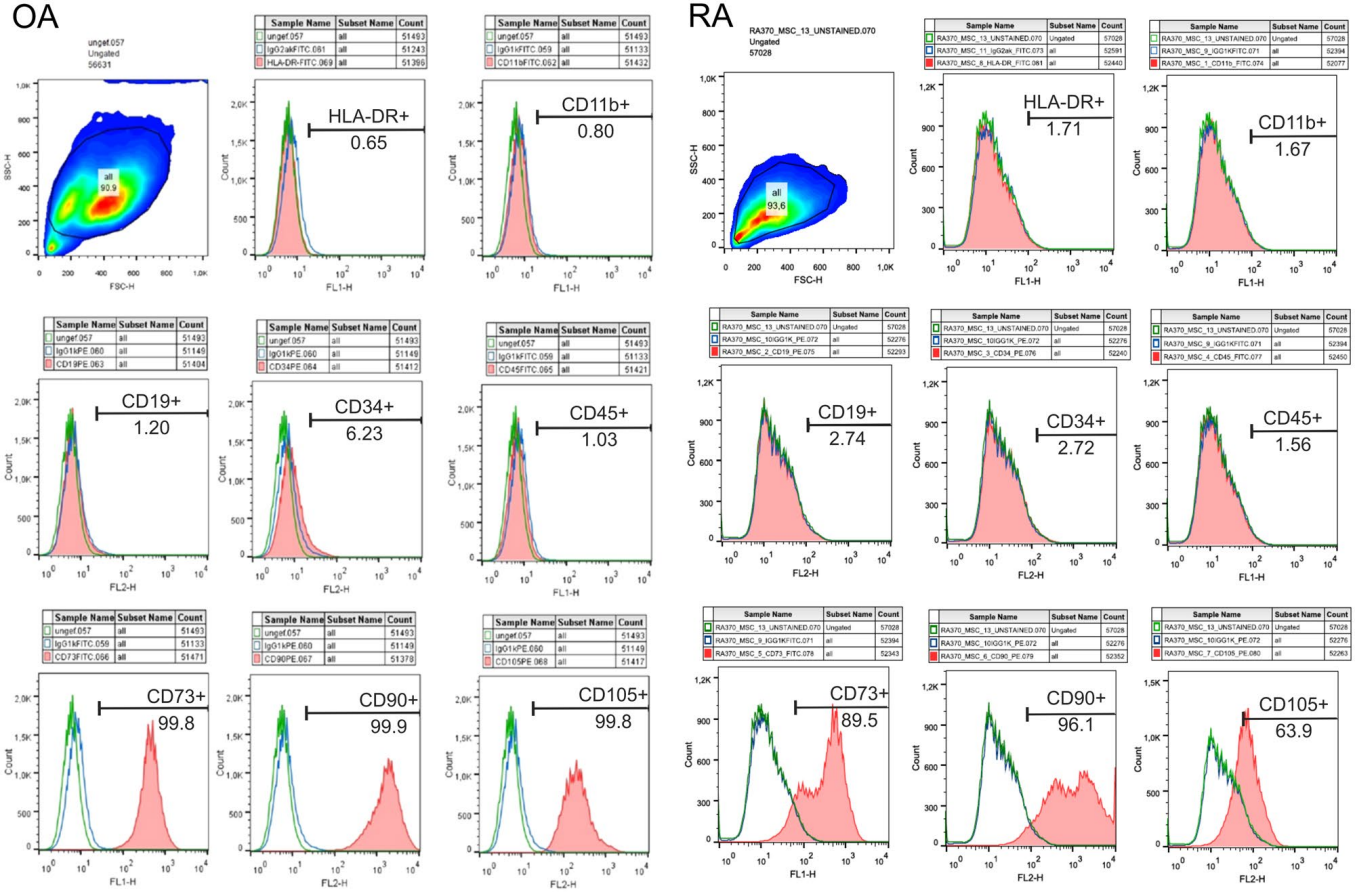
Characterization of MSC-specific surface markers of synovial adipose tissue-derived stem cells (sASCs) isolated from osteoarthritis (OA) and rheumatoid arthritis (RA) donors. sASCs were positive for CD73, CD90, and CD105 and were negative for HLA-DR, CD11b, CD19, CD34, and CD45 as suggested by *The Mesenchymal and Tissue Stem Cell Committee of the International Society for Cellular Therapy*^35^. The red line gives the target surface marker, the green line the unlabeled negative control, and the blue line demonstrates the isotype.

Human synovial adipose tissue was obtained from patients with OA and RA during knee joint replacement surgery (see above). sASCs were isolated as described elsewhere^33,34^. After isolation, cells were expanded in monolayer cultures and stem cell markers were investigated by FACS analysis as suggested by *The Mesenchymal and Tissue Stem Cell Committee of the International Society for Cellular Therapy*^35^. The mesenchymal stem cell characteristics of OA and RA sASCs was confirmed by FACS analysis: sASCs were positive for the surface markers CD73, CD90, and CD105 and were negative for HLA-DR, CD11b, CD19, CD34, and CD45 (Fig. 1)^35^. Passage 3–4 cells were used for catecholaminergic differentiation experiments into the direction of human induced TH+ cells (see next section).

### Generation of human induced TH+ cells (iTH+) and imaging of morphology

Human sASC were plated on poly-D-lysine in flasks and chamber slides (20.000 cells/cm^2^) as described elsewhere^6^. On day 1, catecholaminergic induction was initiated with a specific catecholaminergic medium containing B27 supplement (Life Technologies, Darmstadt, Germany), sonic hedgehog, fibroblast growth factor 8, basic fibroblast growth factor, and on day 9 with additional brain-derived neurotrophic factor (all from PeproTech GmbH, Hamburg, Germany)^6^. The obtained cells were handled as individual cell cultures of each patient.

During 12 days of differentiation, cells were treated with 1 ng/ml or 10 ng/ml TNF (representing a different degree of inflammation). In order to test, whether possible TNF-specific effects during iTH+ cell differentiation might be caused by synovial fluid, we treated OA and RA sASCs during catecholaminergic differentiation with the synovial fluid of the same patient (1 ml synovial fluid/10 ml cell culture medium).

To block TNF-specific effects, the soluble TNF receptor etanercept was used in low and high concentrations (1 µg/ml or 10 µg/ml). Differentiation was performed under hypoxic conditions. Hypoxia was defined as 1% oxygen tension, because inflamed synovial tissue is known to be strongly hypoxic^31^. After 12 days of differentiation, cells were investigated macroscopically and by immunofluorescent staining of differentiation markers such as TH, β-III-tubuline (a neuronal intracellular scaffold protein), vesicular monoamine transporter 2 (VMAT-2), and nuclear receptor related 1 (Nurr1)^5–8^.

Cells with dendritic shape were counted manually by a blinded experimenter using phase contrast images of differentiated cultures (n = 8 RA slides, n = 8 OA slides) and the ratio of dendritic-shaped cells to total cell count was calculated.

For catecholamine determination by HPLC (see below), supernatant was acidified using 20 μl of 0.1 M perchloric acid, and samples were frozen at −80 °C.

### Human mixed synovial cell culture

Synovial tissue preparation and removal of synovial cells was performed as described earlier^36^. Pieces of synovial tissue of up to 9 cm^2^ were excised. One part of the synovial tissue specimen was minced and placed in Dispase I (Roche Diagnostics, Penzberg, Germany). Digestion was carried out for at least 1 hour at 37 °C on a shaking platform. The resulting suspension was filtered (70 µm) and spun at 300 g for 10 min. The pellet was then treated with erythrocyte lysis buffer for 5 min and centrifuged for 10 min at 300 g. The pellet was re-suspended in RPMI 1640 (Sigma-Aldrich, Taufkirchen, Germany) with 10% fetal calf serum.

For stimulation, 50.000 mixed synovial cells per ml were transferred into cell culture flasks. The obtained cells were handled as individual cell cultures of each patient (no pooling). Mixed synovial cells (containing fibroblasts, macrophages, lymphocytes, and dendritic cells as demonstrated in an earlier study, ref.^1^) were incubated under hypoxic conditions in cell culture flasks. Synovial cells were incubated for 24 h with different concentrations and combinations of TNF and etanercept as described above. For catecholamine measurements samples were prepared as described above.

### Immunofluorescence

Cells in chamber slides were fixed with 3.7% paraformaldehyde, underwent drying, and were stored at −20 °C until analyses. For immunofluorescent analyses, cells in chamber slides were rehydrated with 1x phosphate-buffered saline (PBS) for 10 min. After blocking (10% bovine serum albumine, 10% chicken serum, and 10% goat serum), chamber slides were incubated with primary antibodies to TH (AB152; Merck Millipore, Schwalbach, Germany), β-III-tubulin (Abcam, Cambridge, UK), VMAT-2 (Abcam, Cambridge, UK), and Nurr1 (Abcam, Cambridge, UK) at room temperature for 3 h. Primary staining was visualized using Alexa Fluor-labeled secondary antibodies (goat anti-rabbit Alexa Fluor 598 for TH and Nurr1; goat anti-rabbit Alexa Fluor 488 for β-III-tubulin and VMAT-2). Cell nuclei were counterstained with DAPI. Numbers of fluorescently stained cells were counted manually by a blinded experimenter (n = 8 RA slides, n = 8 OA slides) and the ratio of stained cells to total cell count was calculated.

### Noradrenaline quantification

NA in the cell culture medium was assayed by high pressure liquid chromatography with electrochemical detection as previously described by us^37^.

### LDH assay for cell viability

Cell viability was determined by measuring LDH (lactate dehydrogenase) activity in supernatants after stimulation experiments. The assay was performed according to manufacturer instructions (Takara Bio Company, Kusatsu, Japan). The positive toxic control was methanol.

### Data analysis

Numbers of experimental samples investigated are given in the respective figure legends. Data are presented as box plots and as % of controls due to naturally occurring variation in primary cell cultures. Comparisons between two experimental groups were performed using Mann-Whitney-U test. Comparisons between more than two groups were carried out using ANOVA (when data were normally distributed) or ANOVA on ranks (when normal distribution was not given). We applied post-hoc analysis for multiple comparisons according to Bonferroni (SigmaPlot V.11, Systat Software, Erkrath, Germany). P values less than 0.05 were considered significant.

## Results

### TNF effect on cell morphology

**Figure 2:**
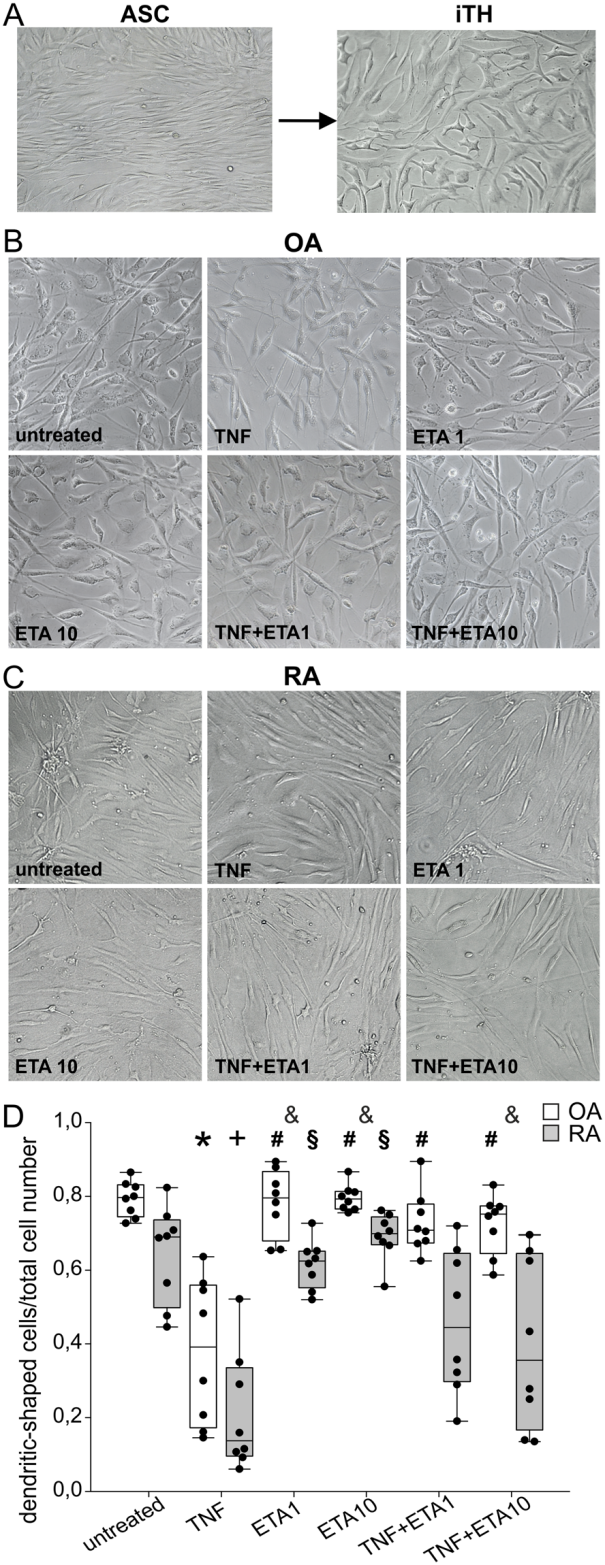
Morphology of generated iTH+ cells. (**A**) Change of fibroblast-like morphology of synovial adipose tissue-derived stem cells (sASC) to neuron-like morphology after 12 days of catecholaminergic differentiation (representative image of OA donor cells of one patient, magnification 200x). (**B**) Light microscopic appearance of OA iTHs after treatment with different concentrations and combinations of 10 ng/ml TNF and 1 or 10 µg/ml etanercept (ETA) (magnification 200x). (**C**) Light microscopic appearance of RA iTHs after treatment with same agents as in B (magnification 200x). (**D**) Number of fully differentiated dendritic-shaped cells after 12 days of catecholaminergic differentiation demonstrated as ratio of dendritic-shaped cells to total cell count (n = 8). Data are presented as box plots with the 10th, 25th, 50th (median), 75th, and 90th percentiles. Each black circle represents an individual patient sample. Significant p-values (p ≤ 0.05) against OA control are presented as “*”; against OA TNF group as “^#^”; against RA control as “+”; against RA TNF group as “^§^”; and differences between respective OA and RA groups as “^&^”. Abbreviations: TNF10 = TNF 10 ng/ml; TNF1 = TNF 1 ng/ml; ETA1 = Etanercept 1 µg/ml; ETA10 = Etanercept 10 µg/ml.

Morphological analysis with phase contrast microscopy demonstrated that iTH+ cells exhibited a neuron-like morphology with branches. In contrast to original fibroblast-like sASC, iTH+ cells formed more intercellular connections after 12 days of differentiation (ref.^5^, Fig. 2A, representative picture).

Regarding iTH+ morphology, we observed qualitative differences between OA and RA: untreated RA iTH+ cells showed more elongated cell bodies and less branching compared to OA iTH+ cells, but cell-to-cell contacts were still visible (Fig. 2B,C untreated). The quantification of cells with dendritic shape confirmed this observation, however, the difference between OA and RA untreated groups was not significant (Fig. 2D untreated).

In OA iTHs and RA iTH+, TNF (10 ng/ml) treatment caused morphological changes, which result in thinning and decreased dendritic shape branching (Fig. 2B). This phenomenon was not observed to the same extent in RA iTH+ cells but was visible, too (Fig. 2C and D, not significant). The difference between OA and RA might be a consequence of the proinflammatory priming of these cells in the tissue before surgery.

Etanercept (in low or high dose) or combinations of TNF plus etanercept did not influence cell morphology in both OA and RA iTH+ cells compared to the respective OA and RA untreated iTH+ (Fig. 2B–D). In general, there was a somewhat higher level of dendritic-shaped cells in OA compared to RA (Fig. 2D), which indicates a better differentiation result in OA cells.

### TNF effect on catecholaminergic differentiation markers in iTH+ cells

**Figure 3:**
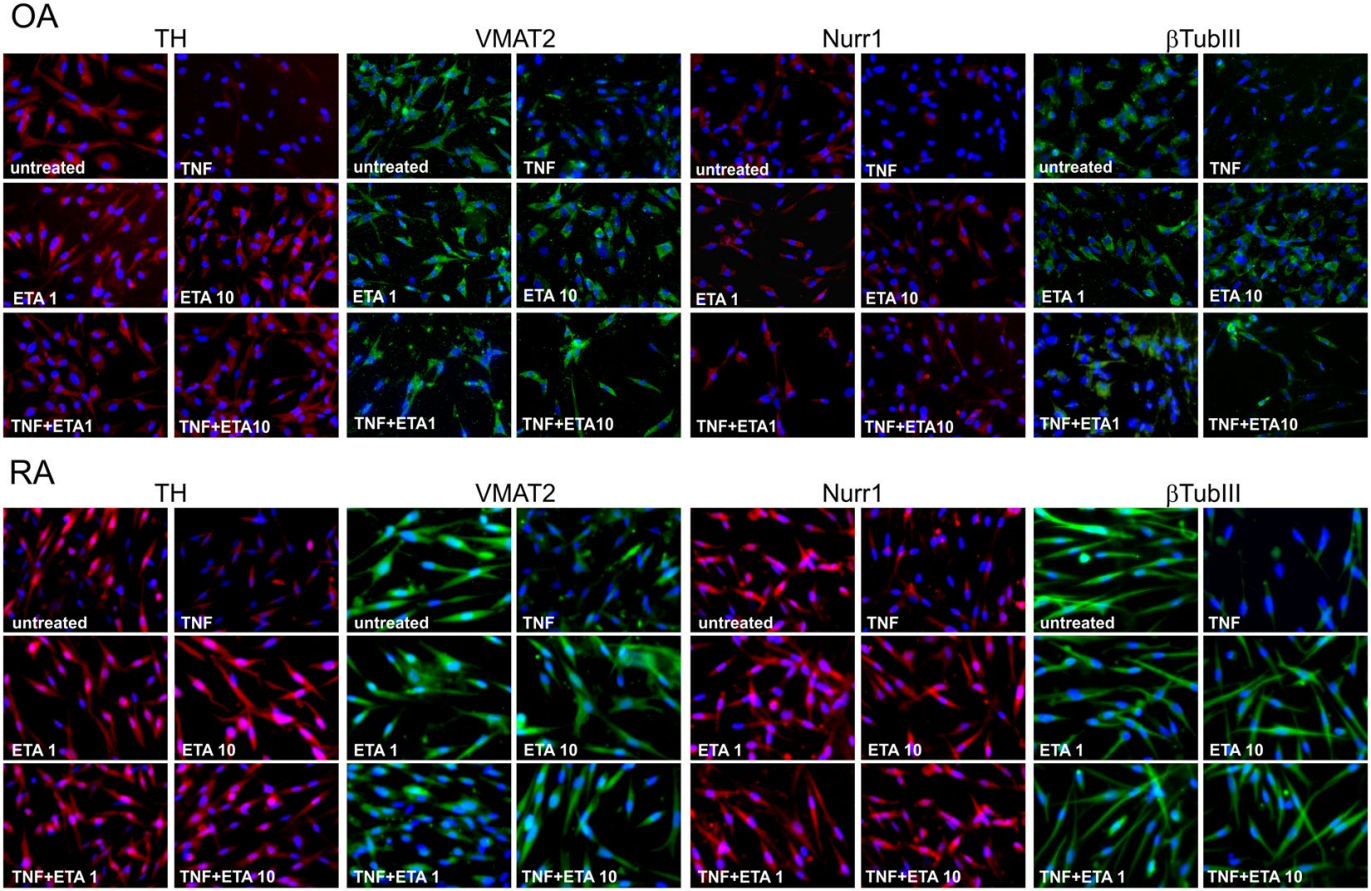
Effects of TNF on catecholaminergic differentiation marker expression. Immunofluorescent detection of tyrosine hydroxylase (TH, key enzyme of catecholamine synthesis), vesicular monoamine transporter 2 (VMAT2), nuclear receptor related 1 protein (Nurr1), and β3-tubulin in OA and RA adipose tissue-derived stem cells undergoing catecholaminergic differentiation treated with 10 ng/ml TNF and/or 1 or 10 µg/ml etanercept (ETA) in different concentrations and combinations (magnification 200x).

Untreated OA and RA iTH+ cells strongly expressed catecholaminergic markers such as TH, VMAT-2, Nurr1, and βIII-tubulin (ref.^5^, Fig. 3). After TNF treatment (10 ng/ml), the expression of these markers was clearly suppressed demonstrated by weaker staining (Fig. 3). The TNF inhibitor etanercept (low and high concentration) alone had no effects on catecholaminergic markers, neither in OA nor in RA iTH+ cells (Fig. 3). However, TNF-mediated suppression of TH, VMAT-2, Nurr1, and βIII-tubulin expression was visibly reversed by both low and high etanercept concentrations in OA and in RA iTH+ cells treated with TNF (Fig. 3).

**Figure 4:**
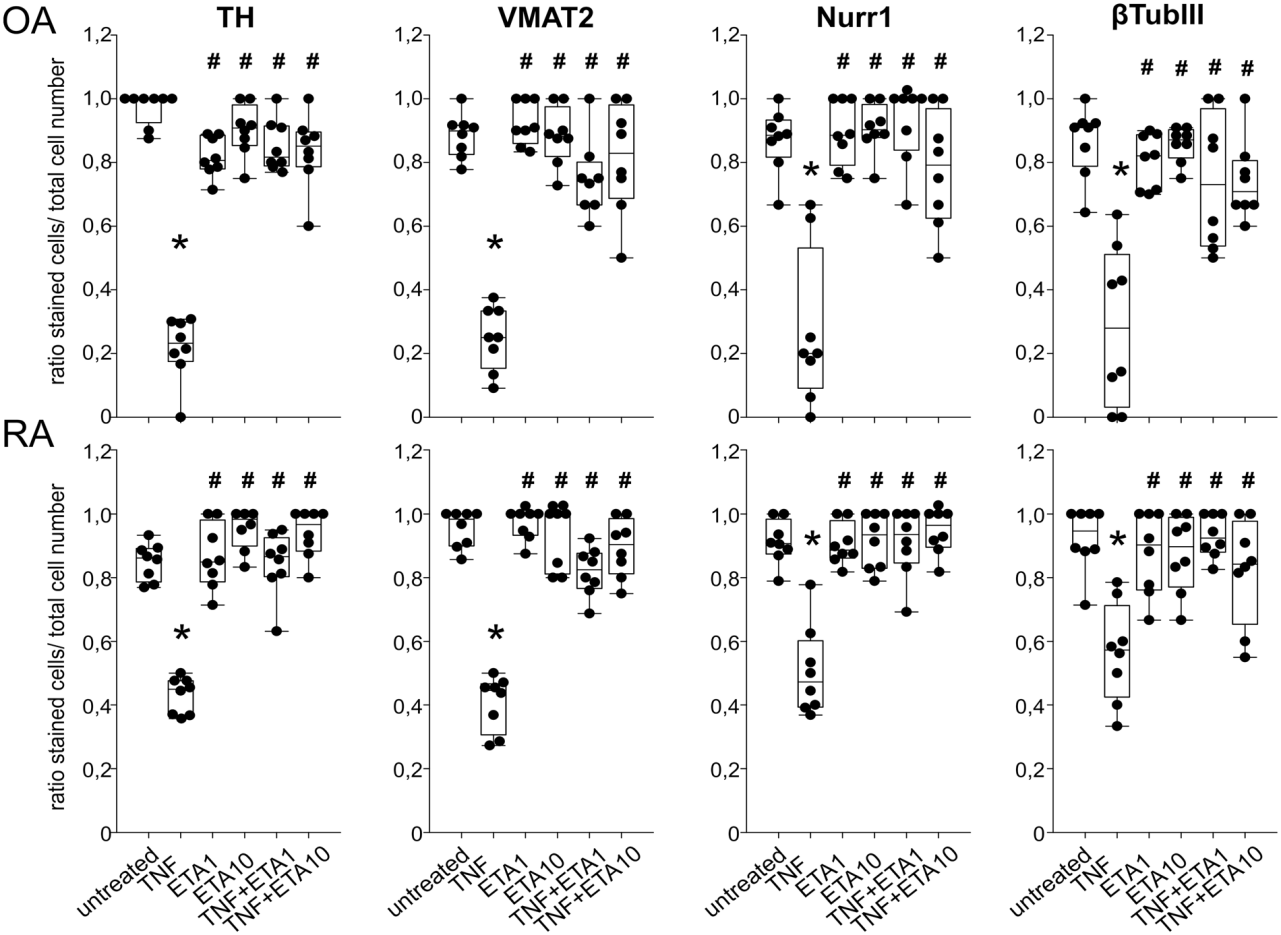
Quantification of catecholaminergic differentiation marker expression. Number of fully differentiated TH-, VMAT2-, Nurr1-, or β3-tubulin-positive cells after 12 days of catecholaminergic differentiation demonstrated as ratio of stained cells to total cell count (n = 8). Data are presented as box plots with the 10th, 25th, 50th (median), 75th, and 90th percentiles. Each black circle represents a patient sample. Significant p-values (p ≤ 0.05) against untreated control are presented as “*”; against TNF-treated group as “^#^”. Abbreviations: TNF10 = TNF 10 ng/ml; TNF1 = TNF 1 ng/ml; ETA1 = Etanercept 1 µg/ml; ETA10 = Etanercept 10 µg/ml.

**Figure 5:**
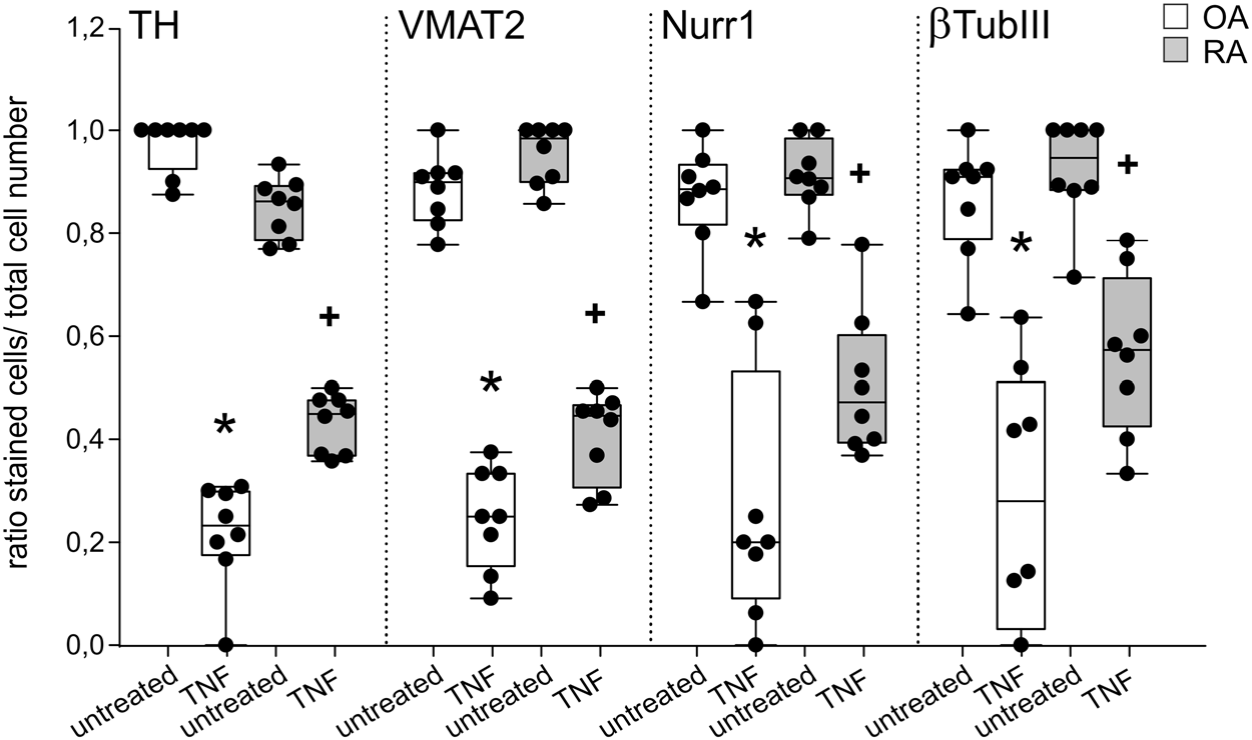
Differences between OA and RA in catecholaminergic differentiation marker expression. Number of fully differentiated TH-, VMAT2-, Nurr1-, or β3-tubulin-positive cells after 12 days of catecholaminergic differentiation demonstrated as ratio of stained cells to total cell count (n = 8). Data are presented as box plots with the 10th, 25th, 50th (median), 75th, and 90th percentiles. Each black circle represents a patient sample. Significant p-values (p ≤ 0.05) against OA control are presented as “*”; against RA control as “+”. Abbreviations: TNF10 = TNF 10 ng/ml; TNF1 = TNF 1 ng/ml; ETA1 = Etanercept 1 µg/ml; ETA10 = Etanercept 10 µg/ml.

All observations for all four catecholaminergic markers were confirmed by quantification of immunofluorescent stainings. In both, untreated OA and RA iTH+ cultures, the ratio of stained cells to total cell count was near 1.0, indicating successful differentiation of the whole cell culture (Fig. 4). In OA and RA iTH+ culture, TNF reduced the ratio of stained to total cells for each catecholaminergic marker (Fig. 4), which was reversed by both low and high etanercept concentrations (Fig. 4). In addition, there were on marked differences between OA and RA cells in a direct comparison (Fig. 5).

Representative control immunofluorescence images with a rabbit IgG isotype are given in Suppl. Fig. 1.

### Effect of TNF and synovial fluid on noradrenaline release during iTH differentiation

**Figure 6:**
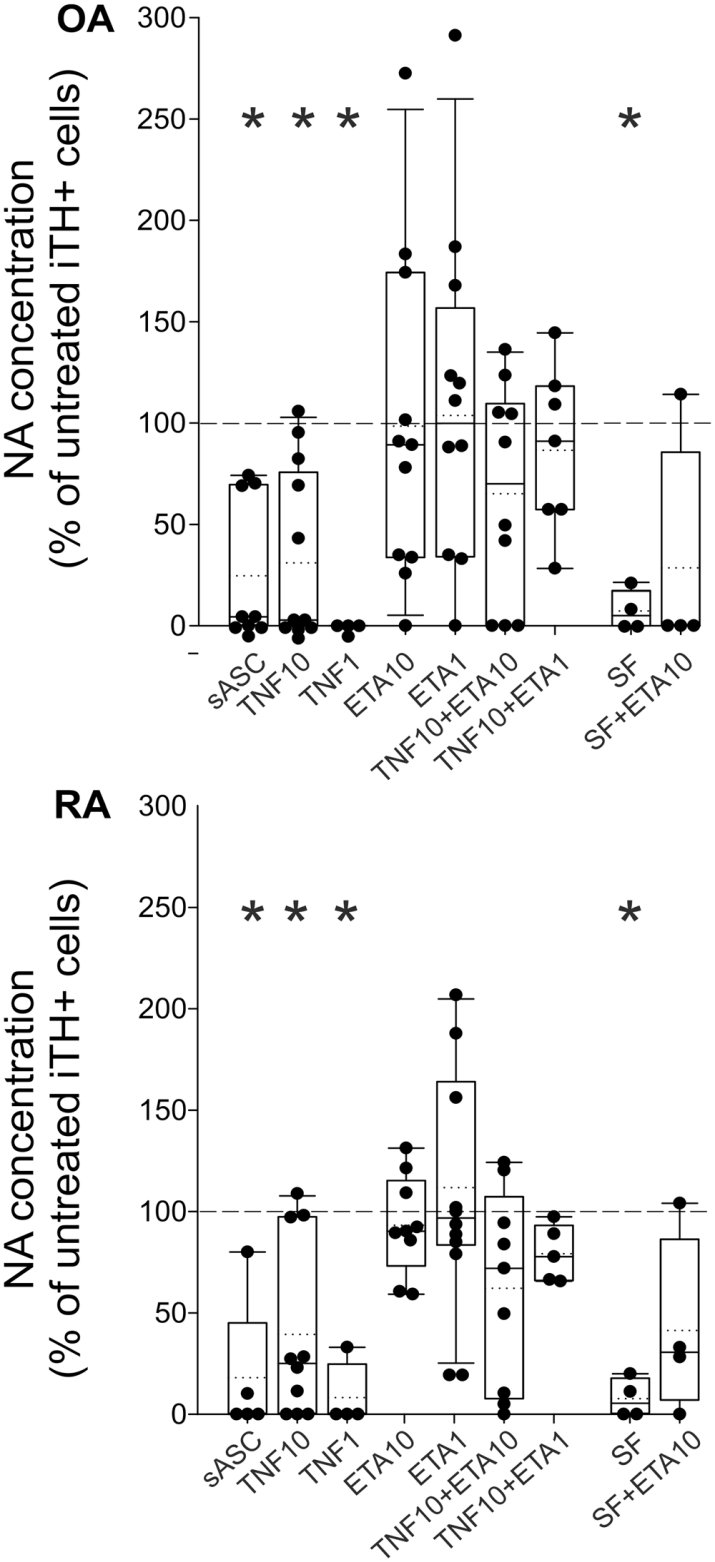
Effect of TNF on noradrenaline (NA) release from iTH+ cells. NA release from catecholaminergically differentiated OA and RA iTH+ cells treated with 10 ng/ml TNF or synovial fluid (SF) with/without 1 or 10 µg/ml etanercept (ETA) in different concentrations and combinations. In all panels, data are presented as box plots with the 10th, 25th, 50th (median), 75th, and 90th percentiles. Values are demonstrated in percent of control (untreated control = 100%, dashed line; n = 4–11; absolute values for negative controls are given in the text). Each black circle represents a patient sample. Significant p-values (p ≤ 0.05) against dashed line control are presented as asterisk. Abbreviations: TNF10 = TNF 10 ng/ml; TNF1 = TNF 1 ng/ml; ETA1 = Etanercept 1 µg/ml; ETA10 = Etanercept 10 µg/ml; SF = synovial fluid.

In supernatants of untreated control cells, NA was detectable in proper amounts (OA: 1.38 ± 2.55 ng/ml; RA: 1.30 ± 3.00 ng/ml; dashed line in Fig. 6). In both OA and RA patients, undifferentiated sASCs synthesized very low amounts of NA (OA: 27.6 ± 69.4 pg/ml; RA: 59.3 ± 127.0 pg/ml), at a level of 10% of control iTH+ cells (Fig. 6). TNF treatment (10 ng/ml and 1 ng/ml) resulted in significantly reduced NA release in RA and OA iTH+ cells (Fig. 6). Etanercept alone did not influence NA release at low and high concentrations, neither in OA nor in RA iTH+ cells (Fig. 6). Etanercept at low and high concentrations normalized the TNF-induced decrease of NA in OA and RA iTH+ cells (Fig. 6).

In order to test, whether TNF-specific effects on NA release during iTH+ cell differentiation might be related to components of synovial fluid such as TNF, we treated OA and RA sASCs during catecholaminergic differentiation with the patient-identical synovial fluid and neutralized TNF. The treatment of RA and OA iTH+ cells with synovial fluid drastically reduced NA release compared to untreated controls (Fig. 6). Etanercept at high concentrations partly reversed the negative effects mediated by synovial fluid, i.e., the measured NA concentrations rose but did not reach the control level (Fig. 6).

### Effect of TNF on catecholamine release in mixed synovial cell culture

**Figure 7:**
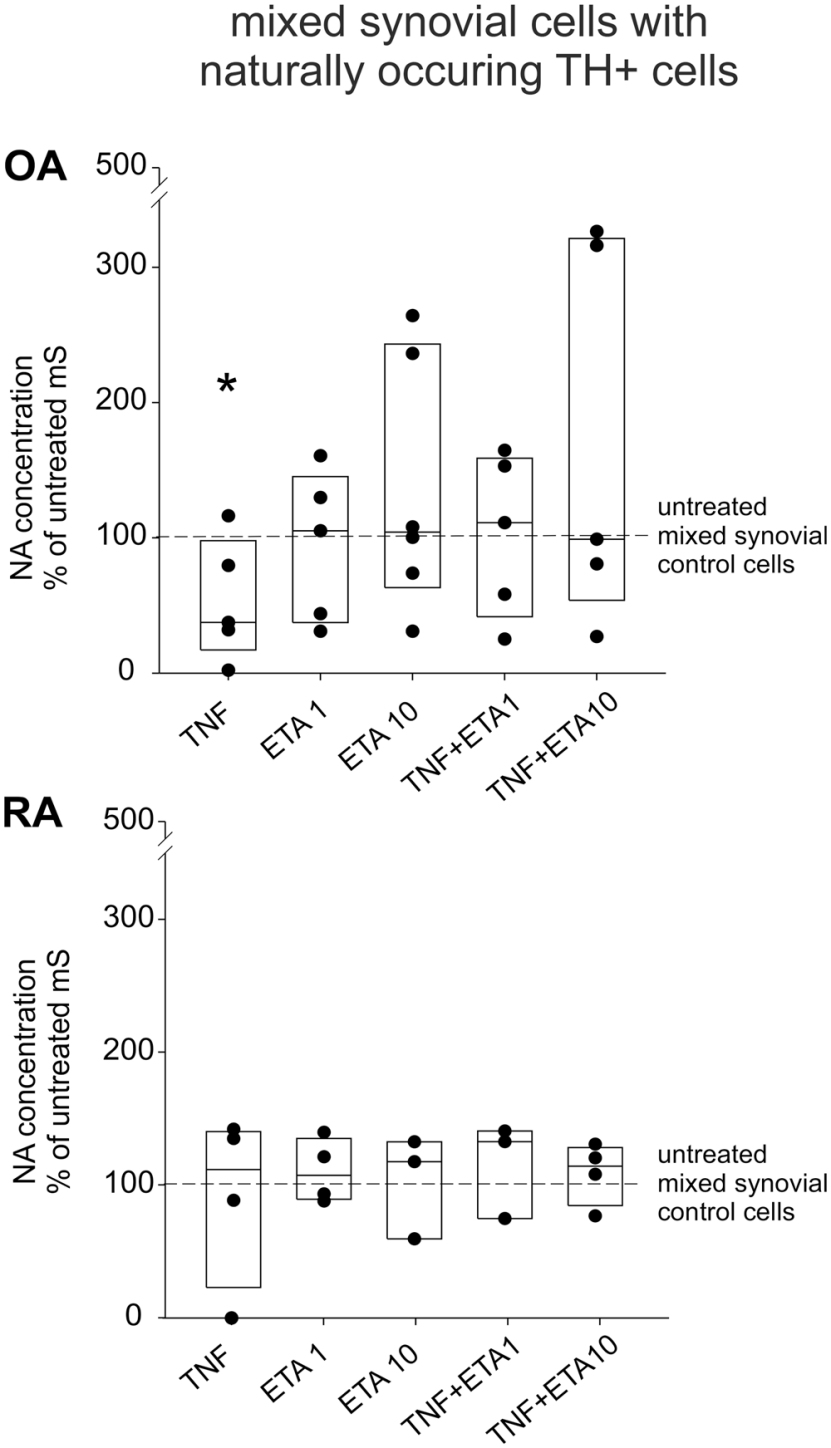
Effect of TNF on noradrenaline (NA) release in mixed synovial cells. NA release in OA and RA mixed synovial cell cultures treated with TNF with/without etanercept at different concentrations and combinations. In all panels, data are presented as box plots with the 10th, 25th, 50th (median), 75th, and 90th percentiles. Values are demonstrated in percent of the control (untreated control = 100%, dashed line; n = 6–9; absolute values for negative controls are given in the text [≈5 × 10^−7^ M]). Each black circle represents a patient sample. Significant p-values (p ≤ 0.05) against control are presented as asterisk. Abbreviations: TNF = TNF 10 ng/ml; ETA1 = Etanercept 1 µg/ml; ETA10 = Etanercept 10 µg/ml.

In earlier studies, we demonstrated the spontaneous presence of catecholamine-producing cells in synovial cell cultures^1,2,5^. In OA mixed synovial cells, TNF (10 ng/ml) significantly inhibited NA release compared to untreated controls (Fig. 7, 84.0 ± 187.5 ng/ml = absolute levels in untreated controls [≈5 × 10^−7^ M]). In OA mixed synovial cells, etanercept alone did not affect NA release, but visibly reversed TNF-mediated inhibition of NA release when applied at low or high concentrations (Fig. 7). In contrast, TNF or/and etanercept treatment of RA mixed cell cultures did not influence NA release when compared to control cells (Fig. 7, 88.8 ± 158.2 ng/ml = absolute levels in untreated controls [≈5 × 10^−7^ M]).

### Effect of TNF and synovial fluid on cell viability

**Figure 8:**
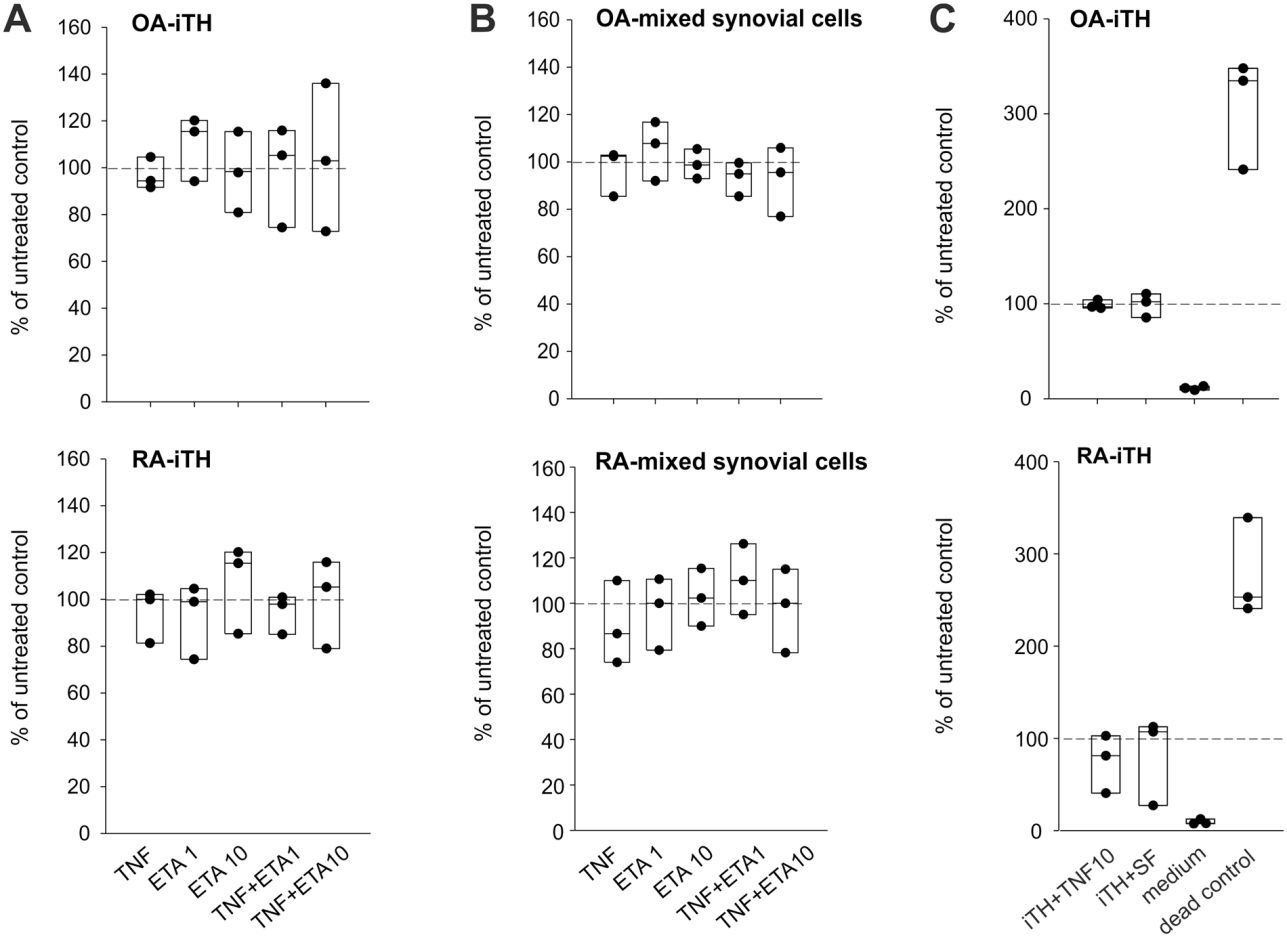
Effect of TNF and synovial fluid on cell viability. (**A**) Lactate dehydrogenase (LDH) release in osteoarthritis (OA) and rheumatoid arthritis (RA) adipose tissue-derived stem cells after catecholaminergic differentiation and treated with 10 ng/ml TNF and/or 1 or 10 µg/ml etanercept (ETA) in different combinations. (**B**) LDH release in OA and RA mixed synoviocytes treated with 10 ng/ml TNF and/or 1 or 10 µg/ml etanercept in different combinations. (**C**) LDH release in OA and RA iTH+ cells after treatment with 10 ng/ml TNF or synovial fluid. In all panels, values are demonstrated in percent of the control (untreated control = 100%, dashed line; n = 3; absolute values for negative and positive controls are given in the text). Each black circle represents a patient sample.

In both, OA and RA iTH+ cell cultures, neither TNF nor etanercept (low or high concentration) or both substances in combination affected cell viability (Fig. 8A). Similarly, in mixed cell cultures of OA and RA patients, no toxic effect of TNF or etanercept was observed (Fig. 8B). Compared to untreated control, synovial fluid treatment similar to 10 ng/ml TNF treatment did not change cell viability in both OA and RA iTH+ cultures (Fig. 8C). In every test, measured optical density in dead control cells ranged between 1.8 and 2.0 units. For this reason, and because calculated relative data are always given as % of untreated control cells, values of dead control cells are only given in Fig. 8C (4^th^ box plot from left). Similarly, cell culture media showed very low optical density values as shown once in Fig. 8C (3^rd^ box plot from left).

## Discussion

Catecholamines are major neurotransmitters of the sympathetic nervous system regulating numerous physiological or pathological processes in the body^38,39^. During RA progression and in the collagen type II model of arthritis, the loss of catecholaminergic tyrosine hydroxylase-positive (TH+) sympathetic nerve fibers occurs and, concomitantly, TH+ single cells with anti-inflammatory character appear in inflamed synovium^1,2,5^. Since NA can have many anti-inflammatory effects through binding to the β2-adrenergic receptor^3,4,40^, the loss of nerve fibers was deemed a proinflammatory phenomenon, while appearance of catecholaminergic TH+ cells can be anti-inflammatory when cell numbers and catecholamine secretion are abundant (high concentrations lead to β2-adrenceptor binding; at approximately ≥10^−7^ M).

Since we and others discussed the use of autologous iTH+ cells as a future therapy in RA patients, further characterization of iTH+ cells is mandatory. One of the main problems in this context is the proinflammatory milieu in RA synovial tissue as exemplified by the presence of TNF^41,42^. In the synovial fluid, TNF concentrations range between 0.1 and 5 ng/ml in RA, depending on infiltrated immune cell number^43,44^. In OA synovial fluid, TNF concentrations are lower (1–10 pg/ml, refs^45,46^). Since TNF has been shown to mediate TH-inhibiting or cytotoxic effects on TH+ cells in brain diseases like Parkinson disease^47^, TNF is an important cytokine that can interfere with catecholaminergic differentiation and catecholamine production. Thus, we aimed to analyze the effects of TNF on catecholaminergic differentiation of iTH  cells of RA and OA patients and NA production.

The starting point of the present work was characterization of OA and RA synovial stem cells from juxtaarticular synovial adipose tissue and the generation of iTH+ cells according to published protocols for mesenchymal stem cells^6–8^. For the first time, human sASC were successfully differentiated into neuron-like catecholaminergic cells. It was shown that the usual fibroblast-like morphology of sASC clearly changed to a neurone-like type of cell with characteristic branches and dendritic shape. In further unpublished studies, we were able to induce axon repulsion of these sympathetic neuron-like cells by semaphorin 3 F, a nerve repellent factor of sympathetic nerve fibers (unpublished results).

Morphologically, differentiation to neurone-like cells was not as perfect as known for healthy mesenchymal stem cells in mice recently performed by us^48^, because these human cells did not form rosette-like structures (small three-dimensional neural tube-like structures in a rosette-shaped form)^49^. There can be several reasons for this phenomenon: first, there might be a decreased potency of OA and RA stem cells to differentiate due to the age of our patients and due to medication^50^. Second, the tissue stems from inflammatory lesions, which might obviate the differentiation process due to milieu factors^51^. Third, the inflammatory process can change signaling pathways and epigenetic signatures in sASC^52,53^, which might be more severe in RA than OA. Nevertheless, the strong immunofluorescent staining of catecholaminergic markers and NA release confirmed catecholaminergic neuron-like differentiation.

In these morphological studies, we observed effects of TNF on OA iTH+ cells in the form of thinning and decreased dendritic shape branching, which was also observed in RA cells. The phenomenon seems to be due to unsuccessful catecholaminergic differentiation caused by TNF. We do not expect that this demonstrates pre-apoptotic effects because cell viability was not affected (LDH assay). This TNF-induced phenomenon was also observed in RA iTH+ cells, although untreated RA iTH+ cells did not show the same dendritic shape as OA cells, possibly because RA cells are already strongly primed by a higher TNF milieu within synovial tissue when compared to OA (RA: 0.1 and 5 ng/ml in RA, refs^43,44^, versus OA: 1–10 pg/ml, refs^45,46^).

One might expect that RA cells are desensitized towards TNF when compared to OA cells. This form of homologous desensitization towards TNF is well known and it can lead to hyporesponsiveness towards TNF^54,55^. Another factor might be medication, which is different in RA versus OA patients. Particularly, glucocorticoids might change the behavior of investigated cells. Due to the numbers of patients that can be investigated in these studies, the influence of medication cannot be studied separately.

Prominent catecholaminergic markers, TH, VMAT2, Nurr1, and βIII tubulin^5–8^ were strongly expressed after differentiation and all were clearly inhibited by TNF treatment. TNF is known to suppress TH expression^16^, which might be indirect through Nurr1 suppression, because Nurr1 directly activates the promoter of the tyrosine hydroxylase gene^56^. At present, no studies exist regarding TNF effects on VMAT2 and βIII tubulin expression so that the demonstrated findings concerning these markers are novel.

TNF-induced loss of catecholaminergic differentiation was particularly visible when looking on NA concentration in supernatants. In differentiated iTH+ cells, TNF-induced inhibition of NA was similar in OA and RA cells, and etanercept reversed these effects. Similarly, patient-identical synovial fluid inhibited NA secretion, which was partly reversed by etanercept. Since reversal with etanercept was incomplete, one expects further inhibitor factors existing in synovial fluid.

In contrast, in mixed synovial cells, NA concentration was markedly reduced in supernatants of TNF-treated OA cells only but not of RA cells. Effects were reversed by etanercept. For different effects in OA and RA mixed synovial cells, homologous desensitization of TNF receptors by TNF and other proinflammatory cytokines might play a role^54,55^. The possible role of medication was discussed above. All effects were independent of cell viability as tested with the LDH assay.

One might argue that concentrations of NA were too low to exert anti-inflammatory effects in our *in vitro* experiments, which are expected to start at equal or greater than 10^−7^ M. The measured concentrations were approximately 10^−8^ M in sASC-derived iTH+ and about 0.5 × 10^−6^ M in mixed synovial cells of patients, where these TH+ cells are already present. For the mixed synovial cells, NA levels would be high enough to exert anti-inflammatory effects. For iTH+ cells, a higher NA concentration can be expected with increased cell numbers in the culture dish. These experiments were carried out *in vitro* under certain culture conditions that yielded the demonstrated results. However, other conditions with higher cell numbers might have shown NA levels up to 10^−6^ M.

In conclusion, although we can generate iTH+ cells from ASCs, which can be a future platform for autologous cell therapy with these cells in RA, TNF and possibly other proinflammatory cytokines in synovial tissue and fluid might disturb the antiinflammatory role of these cells. The data also show that part of therapeutic etanercept effects possibly depend on the protection of naturally occurring iTH+ cells and their NA secretion. It needs to be studied whether once differentiated iTH+ cells can revert their phenotype into a non-catecholaminergic cell when long-term exposed to TNF and other cytokines.

## Electronic supplementary material

suppl. Fig. 1
