# Supplementary material for: TNF inhibits catecholamine production from induced sympathetic neuron-like cells in rheumatoid arthritis and osteoarthritis in vitro
Source: Sci Rep. 2018 Jun 25;8:9645. doi: 10.1038/s41598-018-27927-8 (PMC6018168; doi:10.1038/s41598-018-27927-8)

TNF inhibits catecholamine production from induced sympathetic neuron-like cells in rheumatoid arthritis and osteoarthritis *in vitro*

Markus Herrmann[1], Sven Anders[2], Rainer H. Straub MD[1*], Zsuzsa Jenei-Lanzl PhD[1,3]

[1]Laboratory of Experimental Rheumatology and Neuroendocrine Immunology, Department of Internal Medicine, University Hospital Regensburg, Germany
[2]Department of Orthopedic Surgery, University Hospital Regensburg, Asklepios Clinic Bad Abbach, Kaiser Karl V Allee 3, 93077, Bad Abbach, Germany
[3]Dr. Rolf M. Schwiete Research Unit for Osteoarthritis, Orthopedic University Hospital Friedrichsheim gGmbH, Frankfurt/Main, Germany

***Corresponding author**
Rainer H. Straub
Laboratory of Experimental Rheumatology and Neuroendocrine Immunology
Department of Internal Medicine I
University Hospital Regensburg
Biopark I, Am Biopark 9
93053 Regensburg, Germany
Phone: +49 941 944 7120
Email: rainer.straub@ukr.de

Supplementary Figure 1. Rabbit IgG isotype control pictures of OA and RA iTHs as applied in the context of immunofluorescent staining given in Figures 3.

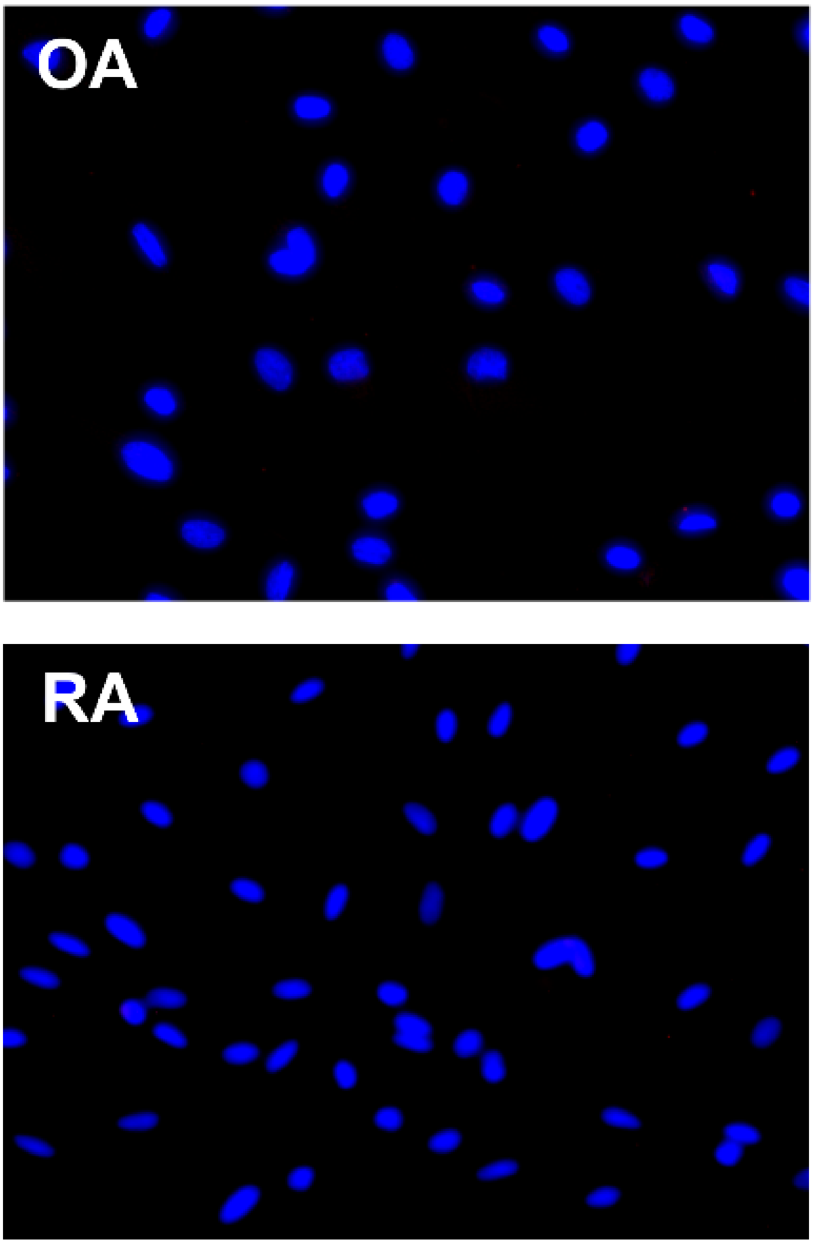

Supplement: Supplementary file 1 — suppl. Fig. 1 [file 41598_2018_27927_MOESM1_ESM.pdf]
